# Multiple submucosal tunneling endoscopic myotomies to address angulation in advanced sigmoid-type achalasia

**DOI:** 10.1093/gastro/goaf103

**Published:** 2026-02-19

**Authors:** Jing-Zheng Liu, Li-Na Fan, Ping-Hong Zhou

**Affiliations:** Endoscopy Center and Endoscopy Research Institute, Zhongshan Hospital, Fudan University, Shanghai, P. R. China; Shanghai Collaborative Innovation Center of Endoscopy, Shanghai, P. R. China; Endoscopy Center and Endoscopy Research Institute, Zhongshan Hospital, Fudan University, Shanghai, P. R. China; Nursing Department, Zhongshan Hospital, Fudan University, Shanghai, P. R. China; Endoscopy Center and Endoscopy Research Institute, Zhongshan Hospital, Fudan University, Shanghai, P. R. China; Shanghai Collaborative Innovation Center of Endoscopy, Shanghai, P. R. China

## Introduction

Per-oral endoscopic myotomy (POEM) is a well-established treatment for achalasia [1–3]. However, its efficacy in advanced sigmoid-type achalasia is limited due to significant anatomical distortion and acute esophageal angulations, which worsen food-passage obstruction [4]. Additional myotomy at these acute angles may help restore the esophagus and improve luminal patency [5].

## Case report

**Figure 1.**
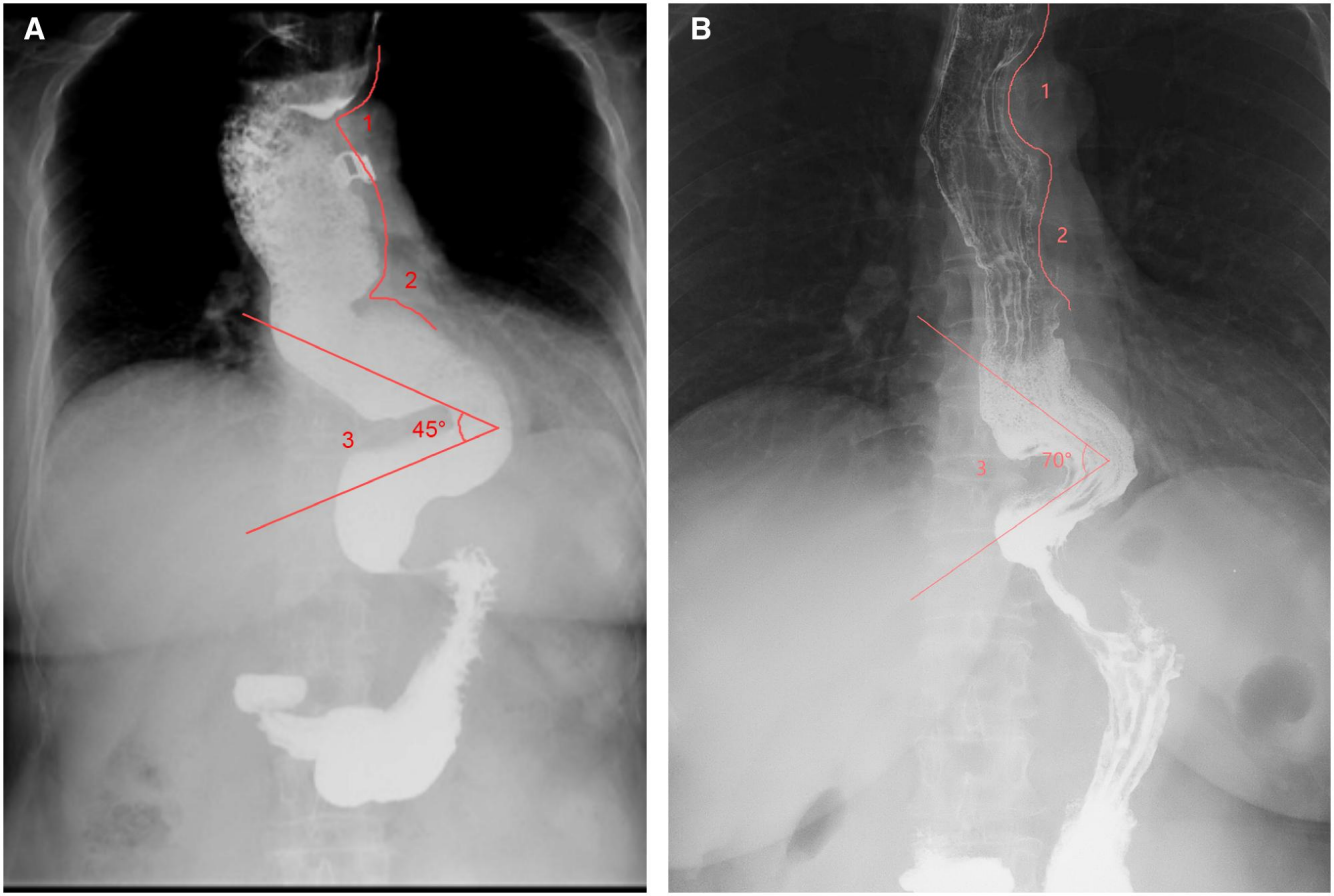
Comparison of preoperative and postoperative esophagograms. (A) Preoperative esophagogram demonstrating three acute angles (labeled with numbers) and a distal beak-shaped appearance. (B) Postoperative esophagogram showing a straightened esophagus and a smooth passage through the esophagogastric junction.

We report a case of a 56-year-old woman with a 10-year history of dysphagia, worsening over the past 2 years. Esophagogastroscopy revealed a dilated, tortuous esophagus with a tightly closed cardia, while a barium radiograph showed three acute angles and a distal beak-shaped appearance (Figure 1A). The gastroesophageal junction was located 45 cm from the incisors. Multiple submucosal tunneling endoscopic myotomies were performed.

The patient was placed in a supine position. After a submucosal injection, the first submucosal tunnel was created between 18 and 27 cm from the incisors. Full-thickness myotomies were performed at two prominent crests formed by the muscular layer, located 20 and 25 cm from the incisors. A second tunnel, spanning between 30 and 48 cm, enabled a long full-thickness myotomy from the acute angle at 32 cm to 2 cm below the cardia, totaling 15 cm in length. The procedure effectively straightened the esophagus and released the cardia. Finally, the entrances of the tunnels were closed by using endoclips and a gastric tube was placed (Video 1). The procedure duration was 60 minutes. The postoperative course was uncomplicated and the patient was discharged on postoperative Day 3. Follow-up evaluations at 4 months, including esophagogastroscopy and barium esophagogram, confirmed the significant improvement in both esophageal obstruction and angulation (Figure 1B and Supplementary Videos S1 and S2).

## Discussion

In patients with advanced sigmoid-type achalasia, persistent failure of low esophageal sphincter (LES) relaxation induces progressive angulation of the esophageal lumen, creating a functional obstruction that impedes bolus transit. This mechanical derangement perpetuates a cycle of luminal distension and architectural distortion, ultimately culminating in sigmoid-like tortuosity of the distal esophagus.

Our case demonstrates that the multiple submucosal tunneling myotomies effectively address both obstruction and angulation in advanced sigmoid-type achalasia. However, morphologic improvement in sigmoid-type achalasia can be a consequence of only LES myotomy, as reported previously [6]. Further studies and long-term follow-ups are warranted to validate the safety and efficacy of this procedure.

## Supplementary Material

goaf103_Supplementary_Data
