# Supplementary material for: Multiple submucosal tunneling endoscopic myotomies to address angulation in advanced sigmoid-type achalasia
Source: Gastroenterol Rep (Oxf). 2026 Feb 19;14:goaf103. doi: 10.1093/gastro/goaf103 (PMC13014363; doi:10.1093/gastro/goaf103)
Supplement: goaf103_Supplementary_Data [file goaf103_Supplementary_Data.zip › Supplementary Video Legends.docx]

**Supplementary Video Legends**

**Supplementary Video 1.** Barium esophagogram performed four months post-surgery.

**Supplementary Video 2.** Esophagogastroscopy performed four months post-surgery.
